# Food fraud and the perceived integrity of European food imports into China

**DOI:** 10.1371/journal.pone.0195817

**Published:** 2018-05-23

**Authors:** H. Kendall, P. Naughton, S. Kuznesof, M. Raley, M. Dean, B. Clark, H. Stolz, R. Home, M. Y. Chan, Q. Zhong, P. Brereton, L. J. Frewer

**Affiliations:** 1 School of Natural and Environmental Sciences, Newcastle University, Newcastle upon-Tyne, United Kingdom; 2 Edinburgh Napier University, Business school, Craiglockhart Campus, Edinburgh, United Kingdom; 3 School of Biological Sciences, Queen's University Belfast, Medical Biology Centre, Belfast, United Kingdom; 4 Departement für Sozioökonomie / Department of Socioeconomics Forschungsinstitut für biologischen Landbau FiBL / Research Institute of Organic Agriculture FiBL Ackerstrasse 113, Postfach, Frick, Switzerland; 5 Food and Human Nutrition Group, Newcastle University International Singapore (NUIS), Singapore; 6 China National Research Institute of Food and Fermentation Industries, National Standardization Centre of Food and Fermentation Industry, 24–6 Jiuxianqiaozhonglu,Chaoyang District, Beijing, P.R. China; Agricultural University of Athens, GREECE

## Abstract

**Background/Aims:**

Persistent incidents of food fraud in China have resulted in low levels of consumer trust in the authenticity and safety of food that is domestically produced. We examined the relationship between the concerns of Chinese consumers regarding food fraud, and the role that demonstrating authenticity may play in relieving those concerns.

**Methods:**

A two-stage mixed method design research design was adopted. First, qualitative research (focus groups n = 7) was conducted in three Chinese cities, Beijing, Guangzhou and Chengdu to explore concerns held by Chinese consumers in relation to food fraud. A subsequent quantitative survey (n = 850) tested hypotheses derived from the qualitative research and theoretical literature regarding the relationship between attitudinal measures (including risk perceptions, social trust, and perceptions of benefit associated with demonstrating authenticity), and behavioral intention to purchase “authentic” European products using structural equation modelling.

**Results:**

Chinese consumers perceive food fraud to be a hazard that represents a food safety risk. Food hazard concern was identified to be geographically influenced. Consumers in Chengdu (tier 2 city) possessed higher levels of hazard concern compared to consumers in Beijing and Guangzhou (tier 1). Structural trust (i.e. trust in actors and the governance of the food supply chain) was not a significant predictor of attitude and intention to purchase authenticated food products. Consumers were shown to have developed ‘risk-relieving’ strategies to compensate for the lack of trust in Chinese food and the dissonance experienced as a consequence of food fraud. Indexical and iconic authenticity cues provided by food manufacturers and regulators were important elements of product evaluations, although geographical differences in their perceived importance were observed.

**Conclusions:**

Targeted communication of authenticity assurance measures, including; regulations; enforcement; product testing; and actions taken by industry may improve Chinese consumer trust in the domestic food supply chain and reduce consumer concerns regarding the food safety risks associated with food fraud. To support product differentiation and retain prestige, European food manufactures operating within the Chinese market should recognise regional disparities in consumer risk perceptions regarding food fraud and the importance of personal risk mitigation strategies adopted by Chinese consumers to support the identification of authentic products.

## Introduction

In recent decades, Chinese domestic food chains have been beset with serious food safety incidents, compromising their integrity and safety, and diminishing consumer trust both internationally and domestically [1]. At the farm level, pressures to increase yields have resulted in the over reliance and use of fertilisers and pesticides to enhance growth and improve the appearance of produce [2–4]. Economic incentives have resulted in opportunities for exploitation, for example the re-labelling and selling of out of date meat products [2, 5, 6]. Many of the high profile food incidents reported by the Chinese media represent cases of food fraud. For example, the Xinhua News Agency reported that, during a 6-month period in 2007, more than 60,000 fake food cases were reported, with over 15,500 tons of substandard food being confiscated, and 180 food manufacturers identified as producing sub-standard food or using inedible ingredients in food manufacturing [7]. A case in point, the 2008 Sanlu melamine poisoning associated with milk used to produce infant formula was responsible for 6 infants’ deaths from renal failure, 54,000 hospitalisations and a further 300,000 cases of illness [8]. Economically, this incident highlighted the inadequate safety standards of food produced in China, significantly affecting China’s performance within export markets and resulting in a global ban on powdered milk products that were manufactured in China [3, 9].

Food fraud is committed when food is deliberately placed on the market, for financial gain, with the intention of deceiving the consumer [10]. Food fraud typically includes “the deliberate and intentional substitution, addition, tampering, or misrepresentation of food, food ingredients, or food packaging; or false or misleading statements about a product”[11]. While these definitions have gained traction within the literature and food policy arena by illustrating the motives and ways in which food can be made “in-authentic”, the emphasis on deliberate and intentional actions highlights external threats to the food chain that infiltrate legitimate food networks [12, 13]. Although organised crime syndicates have been shown to violate global food systems, it can be argued that these definitions direct scrutiny away from legitimate actors within legitimate food chains that can also play a role in the perpetration of food fraud, as was highlighted by the Chinese melamine adulteration case [14, 15]. Whilst framing food fraud in this way maintains industry reputations and protects the public image of a robust and legitimate food system, it neglects the endogenous nature and causality of food fraud. Lord *et al*. [14] provides an alternative definition that better encapsulates the nature of food fraud and its perpetrators in China, emphasising the role of both exogenous and endogenous actors in its perpetration. Thus food fraud is:

”the abuse or misuse of an otherwise legitimate business transaction and an otherwise legitimate social/economic relationship in the food system in which one or more actors undertake acts or omissions of deception or dishonesty to avoid legally prescribed procedures (process) with the intent to gain personal or organizational advantage or cause loss/harm (outcome).”

The intentional deception of consumers differentiates food fraud from food safety incidents. Food safety incidents are characterised as unintentional acts leading to unintentional harm to public health [11].The undisclosed nature of potential adulterants means that food fraud can potentially pose substantial risks to human and environmental health[16]. However, not all cases of food fraud carry public health risks, for example, as is the case in the sale of horsemeat as beef. Fraud of this nature will not impact human health, but it can damage the reputation of brands, and undermine consumer confidence and trust in regulatory institutions, as well as manufacturing and distributive industries to protect the public from potentially unsafe counterfeit and fraudulent products[17]. Little attention in the research literature has been given to consumer’s attitudes towards and perceptions of food fraud specifically. However, much research has been conducted to explore Chinese consumer attitudes towards, and perceptions of, the safety of food. Food safety is identified as being Chinese consumer’s top safety concern [18,19]. Chinese consumers are acutely aware of the risks presented by food safety incidents [20]. Risks that constitute fraud rank as the most significant safety concern, with Chinese consumers reportedly most worried about “counterfeit food” and “inferior quality” food [21]. This finding suggests that Chinese consumers perceive food fraud to represent one of a range of food safety issues, with risk to the safety being a potential consequence of fraudulent activity by actors in the food chain.

In recent decades the Chinese food landscape has undergone significant change. The Chinese economic reforms, which began in 1978, have resulted in liberalisation from a state-regulated food system to a market-based system. Together with the impacts of globalisation, this has improved the variety and availability of food and transformed Chinese food shopping habits and the retail landscape [7, 22]. However, the rate and scale of change has led Chinese consumers to distrust the profit motive introduced into the food supply system, which has arguably increased the opportunity for economically motivated food fraud and consumer deception[7].

In response to the challenges posed by the changing nature of the food system, the repeated occurrence of food safety and quality problems, and to meet the requirements of China’s membership to the world Trade Organization [23], China has been improving governance of its food supply chains [1]. Food safety law and regulation have developed incrementally in China. The first comprehensive reform of the governance of the Chinese domestic food chain was instigated by the country’s first national Food Safety Law which was passed in 2009. Despite significant improvements this made to the regulation of the food system, serious challenges remain [24, 25]. These include scale (regulating huge numbers of small supply chain actors), [26]; fragmented and unclear responsibilities attributable to different supply chain actors, and relatively weak institutions and enforcement [3, 20, 27],the relative immaturity of NGOs and third party certification systems [28], and a lack of data collection and supporting technology [25]. From a consumer perspective, the perceived lack of deterrence of food fraud due to failure to enforce the law, and inadequate penalties for committing fraudulent activities [24]. In response to these recognised shortcomings, revisions to the 2009 law were made, and the second iteration of the countries national Food Safety Law became operational in 2015. The 2015 law expanded and clarified components of the pre-existing framework and gave authorities greater powers to prosecute violators and impose stricter penalties on those contravening the law, making China’s current food safety regulations one of the most stringent globally [27, 29].

A key component of the regulatory reforms has been improved traceability systems and the introduction of product labelling and certification schemes. These represent a key policy tool in China for managing food and regaining consumer confidence in food that is domestically produced [1]. Such schemes are intended to improve the transparency of the domestic food chain and enable Chinese consumers to make objective assessments about the quality of food products [30]. There is no specific certification that addresses food fraud or crime explicitly. However, Chinese food labels now carry multiple certification logos that signify that products meet stringent certification standards and thus infer a level of quality. Food that is domestically produced in China may carry one of three main certifications; ‘Hazard free food’, ‘Green food’ and ‘Organic Food’ [1] as well as country of origin certifications included on foods that are imported into the Chinese market, such as protected designation of origin (PDO) and protected geographical indication (PGI). Such certifications represent one means by which consumers can evaluate the utility of a product and assess whether a product is what it claims to be.

Authenticity provides the mechanism through which the integrity of a product can be objectively assessed. It is the basis upon which products can be differentiated and the means by which the integrity of products can be conveyed, thus supporting consumer decision-making and helping consumers to ensure that products meet their claims. Authenticity has been conceptualised through scientific, policy, commercial and consumer lenses, and, possibly due to this diversity, there is no agreed definition. However, authenticity is typically characterised as requiring two dimensions; i) objective authenticity and, ii) constructivist authenticity [31] summarised in Table 1.

**Table 1:** Dimensions of authenticity.

Dimensions of authenticity	Definition		Example	Consumer protection
**Objective authenticity**	Intrinsic and extrinsic product characteristics that can be proven to be authentic when compared to external criteria [31].		Conforms to official safety or compositional standards and matches the product description provided, preventing consumers from being miss-led, i.e. ISO standards. Verifiable through analytical methods such as stable isotope analysis, DNA analysis, proteomics, lectin chip array and metabolomics [32].	Provides the legislative framework for supporting product authenticity and offers consumer protection through legal recourse.
**Constructivist authenticity**	Constructivist cues are provided by companies to stage the authenticity of their products [31] and are conveyed by indexical and iconic cues.	*Indexical*: Factual, spacio-temporal connection to history [33] demonstrated in the physical attributes of a product.	Verifiable link to a trusted reference point that can be objectively assessed i.e. certifications such as ‘Protected Geographical Indicator (PGI)’, ‘Protected Destination of Origin’ (PDO).	Can be objectively assessed through analytical methods.
		*Iconic*: Reproduction of the physicality of the original product captured in the ‘brand essence’ or symbolic attributes of the product [33].	Conveyed through packaging and imagery.	Non-verifiable, authenticity assessed by how the products congruence with consumers pre-existing knowledge of how the product should be.

Objective authenticity relates to intrinsic and extrinsic product characteristics that can be proven to be authentic when compared to external criteria [31]. An example is provided by the case where production conforms to official food safety or compositional standards and matches the product description provided, preventing consumers from being misled. This evidence-based approach provides the basis for consumer protection and is enacted through the Chinese Food Safety Law 2015. Supporting legislation are surveillance and enforcement activities that are underpinned by a growing range of analytical methods (i.e. stable isotope analysis, DNA analysis, proteomics, lectin chip array and metabolomics) [32]. However, consumers are not readily able to verify the intrinsic and extrinsic nature of products through scientific testing, and therefore rely on constructivist cues provided by companies to stage the authenticity of their products [31]. Companies are able to convey authenticity to consumers through the use of ‘indexical’ and ‘iconic’ cues [33]. A product having indexical authenticity refers to its factual, spacio-temporal connection to its history [34] demonstrated by the physical attributes of a product (i.e. a certification included on packaging such as the PDO or PGI on European that can be objectively assessed). ‘Iconic authenticity’ refers to the reproduction of the physicality of the original product and is typically captured in the ‘brand essence’ or symbolic attributes of the product [33]. Products with ‘iconic’ authenticity possess qualities that hold congruence with consumer’s expectations of an authentic product but do not have an externally verifiable reference point [35]. Iconic cues that can be inferred through packaging represent a general similarity with what the consumer believes the product should be, developed through previous experience with products of that category over time [35]. Thus, a product’s authenticity can only be verified by objective and indexical measures. Whilst a consumer’s ‘iconic’ knowledge of a product supports the identification of authentic products, and are used by manufacturers as an additional means of supporting the identification of authentic products, used alone they are not a basis upon which authenticity can be guaranteed.

Many products feature both iconic and indexical cues, although the focus of most studies has been on Chinese consumers’ use of indexical cues and objective authenticity in relation to identifying safe rather than authentic food. Chinese food consumers have rated “globally recognised certifications” as the most important type of product guarantee and authentication, closely followed by Chinese government certification and brand [36]. Many studies have examined consumers’ willingness to pay (WTP) for food which is of a certified quality, or has certified traceability. Generally, this research has identified a modest WTP price premium for food products of verified quality. Studies identify a number of mediating variables, principally gender, age, educational level, presence of children or old people in the household, income, and knowledge about traceability or the particular certification scheme (e.g. [26]). It is not known whether having health vulnerabilities through for example, age or acute illness, or having responsibility for an individual who has, increases WTP for foods that have been certified. A recent research study conducted by [37] (submitted) which examined Chinese consumers’ willingness to pay for authenticated infant formula milk, found that Chinese consumers mistrust the safety of domestic supply chains, although authenticity cues gave some degree of assurance, especially when associated with third party certification. However, research indicates that a substantial proportion of Chinese consumers do not trust certification schemes, or at least do not trust all of them equally. Bai *et al* [38] report a higher WTP for government or industry certification of milk than for third party certifications, although this was partly dependent on income and education level. Cheng *et al* [39] report evidence regarding counterfeit organic products, fake labels, and corrupt inspection officials from whom certificates may be bought. Additionally, Mol [1] highlights there are numerous organic labels in China, which can be confusing for consumers and undermine the quality inferences that these are intended to convey. Moreover, very few products available to Chinese consumers are covered by certification schemes. Other approaches adopted by consumers to authenticate food include using new “alternative” food provisioning schemes, such as direct sales by farmers or farmer co-operatives, which allow direct information exchange, and establish a high degree of trust between consumer and producer, albeit on a small geographic scale, and which are comparatively few in number [1]. Consumers also adopt individual search strategies to minimise personal risk. This includes buying particular brands and shopping at trusted retailers, including bigger, better known supermarkets [7]. Both Bai et al [38] and Xia and Zeng [40] found that brand and retailer are important factors in consumer decisions with regard to purchasing safe milk. Xia and Zeng [40] found that many consumers (in Beijing) prefer supermarkets not only for their convenience but also the quality of products, with food safety being internalised by large scale chain operators. There was strong brand loyalty as consumers believed that prominent producers are likely to label their products to assure safety to maintain brand value.

Another product attribute which consumers can consider is Country of Origin Labelling (COOL) using the source country of a product or brand as an extrinsic information cue [41]. There is evidence that, for consumer goods in general (as opposed to food specifically), consumers exhibit a preference for imported brands as they are perceived to be modern, novel, prestigious and important for expressing individualism, rather than the traditional values of collectivism. Nevertheless there is some evidence of preference for domestic brands and a rise in local brands across many product categories [41]. The literature contains little evidence regarding consumer attitudes towards imported food, and this topic merits further investigation.

Despite reliance on external cues to guarantee credence factors, including authenticity and food safety, there is evidence that Chinese consumers lack the ability to correctly interpret cues about origin and quality. Kwok *et al* [41] found that, although consumers’ stated preferences were for home-produced goods, their actual purchasing behaviour differed, possibly due to poor consumer knowledge of whether brands are local or foreign. Seitz and Roosen [42] investigated perceptions of Bavarian food products in Bulgaria, Romania, China and South Korea by means of concept mapping and semantic network analysis. The semantic networks for China were far less complex than for the other 3 countries, suggesting very low knowledge of product categories. Zhu *et al* [43] examined motivations of purchasing behaviour by Chinese tourists in Europe who purchased not only luxury goods, but functional commodities such as milk powder. The authors suggest that most study participants lacked a clear understanding of the real meaning behind the international brands, although, branding was symbolic of high quality.

Despite considerable reform of the governance of the food system in China, Chinese consumers perceive there to be numerous risks associated with the purchase and consumption of food that is domestically produced. In order to improve consumer trust in the Chinese domestic food supply chain and food that is produced in China, it is important to understand consumer perceptions of the risks posed by food fraud. Constructed cues of authenticity are an important mechanism used as a heuristic (or decision rule), which consumers can use to establish the authenticity, quality and/or safety of a product, thus increasing consumer trust. From an industry perspective, it is therefore important to understand which credence attributes and cues of authenticity are preferred and used by Chinese consumers to support decision-making, attitude formation and intention to purchase. Thus, this research had two main aims;

1. to qualitatively explore Chinese consumer’s attitudes towards food fraud;
2. based on the results of the qualitative research, and the scientific literature, to quantitatively explore the relationship between consumer’s beliefs and attitudes to food products in the supply chain and intention to purchase authentic European food products.

Recognised globally for their high level of production quality and safety standards, European food products were selected as the focus of this research as they provided a comparator to products produced in China. Moreover, there are considerable associated risks to the reputation of the European food chain of food adulteration and fraud, and notable reputational benefits to European food manufacturers of demonstrating the authenticity of their food products [44]. As Europe’s second largest export market (after the USA) the Chinese market represents significant market potential to European companies operating and exporting food and drink to China [45]. Consumer focused research will therefore support European companies to position and differentiate their products in this market.

## Methods and results

The research adopted a two-stage approach. As the intended target market for imported European Food products, affluent middle-class Chinese consumers were the foci of this consumer focused research, and hereafter referred to as ‘Chinese consumers’. In the absence of prior research regarding Chinese consumer attitudes towards food fraud, and in order to provide contextual understandings, the first stage of this research used an exploratory qualitative methodology. Focus groups were conducted to explore Chinese consumer’s attitudes towards food fraud, the perceived risk this posed to consumers, the psycho-social determinants of Chinese consumer trust in the domestic food supply chain and the strategies employed by Chinese consumers to ensure the integrity of the food that they purchased and consumed. The second phase employed a quantitative survey, drawing on the focus group results and insights from the scientific literature. The survey permitted hypotheses established in the qualitative phase to be tested, and focused on identifying the factors influencing consumer attitudes towards, and intention to purchase, authenticated “European” foods. The influence of geographical location on attitude and purchase intention were also explored. Ethical approval for the protocol of both studies was reviewed and approved by the lead authors institutions Ethics Committee in August 2014.

### European food and drink product foci

Three product categories; i) infant milk formula (IMF), ii) Scotch whisky, and iii) olive oil, were the foci of this study (see Table 2). The products were selected due to the contrasting perspectives they lent to exploring authenticity which are represented by the products’ differing consumption occasions, target consumers and susceptibility to fraudulent activities in China.

**Table 2:** European food and drink product research foci.

Product	Incidents	Insights
**Infant milk formula (IMF)**	Melamine Scandal China (2008) 53% of Chinese infants are ‘bottle-fed’ and demand for infant formula likely to increase following reform of China ‘one child policy’ [46].	Provide insights into the importance of; • Country of origin and traceability (which may include factors such as labels, packaging, certifications, country of origin, brand, retail outlet and price) in the purchasing decision • The authenticity and integrity of a product category used by a ‘vulnerable’ target group (i.e. babies and young children), who are reliant on the product as a primary source of nutrition and whose healthy growth requires a nutritionally sound and safe food source.
**Olive oil**	Gutter oil (2011) [47]. Gutter oil, or illicit cooking oil that has been recycled from waste oil collected from restaurant fryers, drains, grease traps and slaughterhouse waste from “table to table” [47]. Adulteration of premium olive oils with lower grade alternative oils and there have been public mis-description scandals relating to this	Olive oil will support analysis of issues relating to: • Traceability, including an understanding of labels, packaging, certifications, country of origin, brand, retail outlet and price. • The importance of authenticity where ‘health’ is an important attribute.
**Scotch whisky**	Aspirational and novel product profile has resulted in its implication in food fraud. ‘Scotch Whisky has protected geographical indication (GI) status in European law (EC Regulation 110/2008)’ which is also recognised in Chinese legislation and has provided the legal basis for the Scotch Whisky Association to work with Chinese authorities to prosecute fraudulent activities on behalf of its members [48].	Scotch whisky provides an opportunity to explore: • The importance of country of origin and GI status as an indicator of authenticity • Consumer concerns about the purchase and consumption of a high value product where counterfeiting has been demonstrated.

### Study 1: Focus groups

Focus groups are a well-established technique for providing a range of views, which are enhanced by group interaction and have particular utility in situations where little is known about a topic or context in which decisions are made [49] In the absence of prior qualitative research exploring Chinese consumer attitudes to food fraud, the focus groups aimed to develop detailed baseline understandings of Chinese consumer attitudes towards food fraud. This was explored, together with their strategies to ensure the food that they purchased and consumed was of the nature, quality (and safety) demanded and the importance of improving the integrity of the domestic food supply chain. The findings informed the development of quantitative data collection tool, and subsequently supported the interpretation of the data analysis.

Focus groups were conducted in three Chinese cities (Beijing, Guangzhou and Chengdu) represented tier 1 and tier 2 urban conurbations located in the north, south and west of China respectively. Tier 1 cities are the most economically developed cities in China and are associated with the most affluent consumers nationally. Tier 2 cities are developing economically, with consumer trends tending to follow those in tier 1 cities. Two discussion guide protocols were developed to account for the differences in usage profiles and contexts of the focus products. The discussion guides were identical in design and the questions asked in each guide were intentionally the same, although different product prompts were used to stimulate discussion around the questions, thereby permitting comparative analysis across the products and groups. The first protocol used IMF and olive oil, and the second Scotch whisky and olive oil as prompts. The discussion guides followed a ‘funnel’ approach [50], starting with broad consideration of how participants selected food and any associated concerns. This lead into specific consideration of participant’s conceptualisation of food authenticity and experiences of food fraud. In this instance, product prompts were chosen on the basis that they were; i) all available to purchase in the domestic Chinese market, and ii) represented as either; a) imported European products, b) European branded products that had been produced in China, or c) products that were produced and packaged in the domestic Chinese market. Physical examples of IMF and olive oil and pictures of Scotch whisky were used as discussion prompts. Focus group discussions closed by considering the perceived importance of improving the integrity (i.e. the reliability, trustworthiness, transparency, morality and ethical conduct) of actors and stakeholders in the domestic food supply chain.

#### Sample

Seven focus groups (including one pilot group), consisting of a total of 42 middle-class Chinese consumers, (based on income and education level), and representative of the identified target markets of the 3 product groups (Table 3), were conducted in Beijing, Guangzhou and Chengdu, China. All participants were the main or joint decision maker for food purchasing in their home, and have resided in their respective city for 3 years or more. The sample included equal representation of gender (n = 22 male, n = 20 female) and all participants were aged between 18–45 years (Table 3).

**Table 3:** Focus group sample characteristics.

Focus Group location and theme	Focus Group number	Participant no.	Participant code	Age	Gender	Monthly Income (RMB ¥)	Weekly food spend (RMB ¥)
**Total**	n = 7	n = 42	-	22–48	n = 22 male n = 20 female	10000–40000	1401–3500
**Beijing:** **Scotch whisky and olive oil**	1	n = 6	Beijing G1 male Beijing G1 female	22–42	n = 4 male n = 2 female	15000–40000	1400–3000
**Beijing, Scotch whisky and olive oil**	2	n = 6	Beijing G2 male Beijing G2 female	24–48	n = 2 male n = 4 female	15000–30000	1400–3000
**Beijing, IMF and olive oil**	3	n = 6	Beijing G3 male Beijing G3 female	35–42	n = 4 male n = 2 female	15000–40000	1400–3500
**Chengdu, Scotch whisky and olive oil**	4	n = 6	Chengdu G4 male Chengdu G4 female	24–37	n = 2 male n = 4 female	10000–16000	1200–1800
**Chengdu, IMF and olive oil**	5	n = 6	Chengdu G5 male Chengdu G5 female	25–36	n = 4 male n = 2 female	11000–20000	1200–2500
**Guangzhou, IMF and olive oil**	6	n = 6	Guangzhou G6 male Guangzhou G6 female	29–36	n = 4 female n = 2 male	16000	1400–2000
**Guangzhou, Scotch whisky and olive oil**	7	n = 6	Guangzhou G7 male Guangzhou G7 female	29–36	n = 4 male n = 2 female	16000–18000	1400–2000

All data were collected in January 2015 by Social Science Research Agency Millward Brown (MB). Discussion guides developed by the authors were translated by MB into Mandarin, then back-translated to ensure meaning. All focus groups were conducted in Mandarin by the same moderator and simultaneously translated into English for benefit of the research team, who observed all focus groups. This supported their understandings of what was being said (for example, through observation of group dynamics), and to minimise information loss in the subsequent transcriptional translational process of the qualitative data. Each group lasted approximately two hours and was audio recorded to aid verbatim transcription and subsequent data analysis. Transcripts were translated into English for analysis. In recognition of their contributions to the research participants were provided with a small incentive payment.

#### Data analysis

Qualitative analysis was conducted using Nvivo 10 [51] and followed a three-stage process. First, transcripts were open coded independently by two coders using an inductive, grounded approach [52]. Key concepts and categories were discussed and an initial coding framework developed. Next, the coding framework was refined iteratively by both coders to develop an exhaustive list of mutually exclusive codes. Finally, the full data set was coded into the framework.

#### Focus group results

The focus groups revealed a broad range of insights that were categorised into five main themes relating to: 1) food fraud as a perceived food safety hazard; 2) the psycho-social determinants of trust in the domestic food supply chain; 3) the perceived risks of food fraud for the consumer; 4) the cues of authenticity used by consumers to support decision making and ensure the integrity of the food they purchased and consumed; and 5) the perceived benefits for consumers of demonstrating authenticity. The findings of the qualitative work presented here are those that contributed to the development of the conceptual model for empirical testing in the questionnaires in stage two (Extended qualitative results are available as a report [53]). For brevity, the findings are summarised in Table 4 and the accompanying narrative explanation below. The presentation of the results reflects the stages of the thematic analysis process. It identifies the inductively derived and abstracted theme title, its definition (subsequently used to support the coding process), and supporting verbatim participant quotes as illustrations of the emergent theme (direct translations from Mandarin to English) and together with the analytical interpretation of the data by the researchers which raised the analysis to the level of abstraction. The findings presented here summarise the perceptions and attitudes of the Chinese participants included within the qualitative study. It is notable that their interpretation of food fraud and consequences are based on their attitudes and perceptions and may differ from broadly accepted ‘expert’ definitions.

**Table 4:** Focus group discussions: Emergent themes supporting evidence and researcher interpretations of the data.

Theme number	Theme	Theme description (derived from qualitative focus group transcripts)	Evidence	Researcher interpretation of the qualitative data
1	**Food fraud as a food hazard concern**	The fraudulent food practices identified by the participants were associated with perceived food safety risks i.e. melamine in milk.	‘I just cannot trust the food in China’ (Chengdu, G4, male). ‘I think these are all worries for the food we eat, for the meat, there is lean pork powder, using the pork to replace the beef, or the meat that is from ill animals not intended for human consumption’ (Guangzhou, G6, female). ‘I am afraid that the imported milk sources from abroad have experienced second packaging and domestic packaging, which will add something we don’t know, second pollution.’ (Chengdu, G5, male). ‘The news have reported, that vegetables have been grown with pesticides and hormones that are over the limited amount’ (Guangzhou, G6, female). ‘Lots of ingredient are not listed, but the ingredient which are not in the list may cause bad effects on body’ (Chengdu, G4, male). ‘I am also worried that there are too much imitation products particularly those in small enterprises’ (Chengdu, G4, female). ‘It is true. However good the package is or how many certifications it has, it can be fake.(Chengdu, G4, male).	Chinese consumers considered fraudulent practice to be present within the Chinese food system. Food fraud was perceived to occur as a result of fraudulent practices by food chain actors (i.e. adulteration, tampering, counterfeiting). Consumers identified fraudulent practices to represent a possible risk to the safety of food. Food safety concerns were included within the majority of examples of food fraud provided by participants. This suggests that consumers may not distinguish between the risks posed by food fraud and food safety incidents arising from non-fraudulent causes.
2	**Perceived risk**	Fraudulent practices as perceived by the participants and how these link to their food safety risk perceptions.	‘I feel nowadays that many people [food producers] have abused the use of hormones and chemicals, that have a bad effect on the body and health’ (Chengdu, G4, male). ‘I felt very uncomfortable, and do not know whether my body will have any problems or not’ (Guangzhou, G6, female). ‘Now some foods are exposed to public that they have toxins which have bad effects for the family and children’ (Chengdu, G4, female). ‘The baby cannot distinguish what they have eaten, so their foods need to be checked. We would rather maltreat ourselves than maltreat our children’ (Chengdu, G4, female).	Food which had been subjected to fraudulent activity was perceived to carry risks to the safety of food (see Theme 1 above). The potential for negative impacts to health were the most significant risks identified by participants, which were perceptually associated with food fraud. The perceived risk of harm at the level of the family, and to children was linked to consumer concern specifically.
3	**Structural trust**	Consumer trust in the food system and associated actors and stakeholders. Actors and stakeholders were identified by participants to include industry, regulators and enforcement agencies.	‘[The government] ‘just can’t control this [food fraud] problem’ (Guangzhou, G5, female). ‘[it] [food fraud] must be accept[ed] if you must live here’ (Chengdu, G4, female). ‘Chinese people [actors in the food industry] have faith about money, they believe profit is the most important thing’ (Beijing, G1, female). ‘So they just put things to the first-tier city in China, but it seems like the development of supervision for the countryside in comparison to the city is not good, one reason is they have no money.’ (Chengdu, G5, male).	Chinese consumers did not appear to trust food chain actors, and hence the food system. Chinese consumers indicated that they did not trust governance structures, and organisations tasked with protecting consumer interests in relation to food. Lack of trust was perceived to be underpinned by a number of factors including; the scale and complexity of the Chinese food system; the pursuit of profit by food chain actors; and the perceived lack of transparency regarding the food system and how it was governed.
4	**Authenticity cues**	These are the indicators consumers identified which they used to support judgements about perceived authenticity (including quality and safety) of foods.	‘We try and buy better raw materials, or a great brand or buy raw materials that has a high price. We can feel comfortable this way’ (Beijing, G3, female). ‘The most important thing is that my friends have no problem after eating. Then I check information online’ (Chengdu, G5, female). ‘I think it is more reliable for imported food, especially the developed countries in the EU, they have higher standard of product quality, so they are more reliable than products that are made in China’ (Guangzhou, G6, male). ‘It is hard for consumers to judge which kind of product is safe. We try to buy from big brand and big supermarket…We can only tell from the way we buy. We cannot search everything we buy from internet. We eat food everyday. It is really hard for us to judge everything we buy. (Chengdu, G4, male). ‘Famous brands are safer’ (Chengdu, G3, male). ‘If it is for the wine or milk, I will certainly go to Hong Kong and Macao’ (Guangzhou, G7, male) ‘We can only buy at imported supermarkets…, whose products are safe although a little expensive…. Although it is more expensive than other supermarkets, its quality can be assured.’ (Chengdu, G4, female).	Consumers reported adopting different “risk relieving” strategies pre- and post- consumption, to help them make judgements regarding the authenticity of food and to mitigate the perceived risks (associated with poor quality and safety) associated with food subjected to fraudulent practices. The use of indexical (PDO, country of origin labels, ISO standards, Hazard Free, Green Food), and iconic authenticity cues (i.e. product packaging, labelling and imagery) represented one component of a broader risk relieving strategy that also included extensive product information search (i.e. use of social media, internet search, personal recommendations), carefully selected food acquisition sources (i.e. online and physical retail outlets) and reliance on a range of “domestically situated” practices (i.e. washing foods to remove residues, growing fruits and vegetables).
5	**Perceived benefits of demonstrating authenticity**	Consumers’ expressed requirements in relation to producer demonstration of authenticity.	‘We hope those food system and supervision can be strengthened. We ‘consumers’, are actually reactive. We can only look forward to the supervision and food safety incidents can do stricter controls.’ (Chengdu, G4, female). ‘At least 99% of consumers will believe that it is real. We will feel strong confidence when we buy products. If the enterprise can do that [provide traceability assurances]’ (Beijing, G3, male). ‘If the supervision becomes strict, things won’t be like this’ (Guangzhou, G6, female). ‘I think it’s quite worrying to live in China, but I think change is needed to change our attitude’ (Guangzhou, G6, female). ‘I want the enterprise to fight fake products… because the department in government do nothing’ (Chengdu, G5, male). ‘If there is some way to show it [authenticity] to the consumer [it will] add to our trust, which means we can be more relieved when we… buy’ (Guangzhou, G6, male).	Greater measures and communicative actions on the part of food chain actors and stakeholders were required by consumers to reduce the perceived risks to consumers of encountering food fraud, and to improve consumer confidence and trust in the domestic food supply chain. Specific activities described by consumers as relevant included the demonstration of authenticity through a range of regulatory and enforcement activities, and enhanced by transparency *via* improved traceability and accountability through the enforcement of penalties. Greater consumer recourse when food did not meet with quality and/or safety expectations and improved communication between food chain actors and stakeholders, and consumers, were consumer requirements in relation to producer demonstration of authenticity.

The most salient theme to emerge across the groups was the perceptual link between food fraud and food safety. Embedded within the examples of food fraud incidents provided by participants were food safety concerns, for example, the deliberate overuse of chemicals in production, or the intentional adulteration of products with harmful substances. The nature of historic incidents of food fraud (most notably the melamine in powdered milk scandal) and the considerable impact of these to public health resulted in a general perception amongst consumers that food fraud is a potential food hazard, included as one of a range of issues that represented a risk to the safety of food (Theme 1). The most significant risk to the consumer associated with fraudulent practice in the food chain was the potential risk this posed to consumer health (Theme 2). Whilst consumers acknowledged the risk this posed to Chinese society generally, risk perceptions were concentrated at the level of the family unit, and the greatest concern was shown for the potential long-term cumulative impacts on the health of infants and children. The pervasive nature of fraud within the Chinese food system had significantly reduced consumer trust in food that was domestically manufactured. A lack of structural trust, characterised by low levels of trust in actors and stakeholders in the food chain and mechanisms for its governance, was observed across all groups (Theme 3). Regional differences across China in the governance of the food supply chain was recognised in all groups, with participants acknowledging that regulatory activity tended to focus on tier one cities, such as Beijing, Shanghai and Guangzhou with little support given to lower tier cities and rural areas which were considered to be the locations most vulnerable to food fraud. Participants residing in Guangzhou commented on the city’s geographical proximity to Macau and Hong Kong where food safety standards were perceived to be superior to those in mainland China. Participants in Guangzhou reported to travel to these territories to purchase in order and ensure the authenticity of food products. The lack of trust in the food chain was isolated to domestic supply chain, with greater levels of confidence shown for international food systems. Across all groups, the European food supply chain was regarded as offering greater levels of authenticity, quality and safety assurances. In response to the perceived food risk that food fraud represented, and the low levels of trust in the Chinese food system, consumers had developed coping mechanisms to the dissonance arising from food fraud (Theme 4). These ‘risk-relieving’ strategies were employed by consumers to ensure that food was of the nature and quality demanded. Strategies included careful selection of acquisition sources and (where possible) seeking imported food products, particularly those produced in Europe. Indexical and iconic cues provided by food manufacturers to denote authenticity were used to support the identification of identify authentic products. Despite producers providing a range of cues of authenticity to the consumer, consumers did not explicitly distinguish between attributes intended to infer a products safety, quality and reliability although these were implicit within discussions and considered holistically when making assessments of product integrity. Traditional indicators such as country of origin, price and brand, and physical prevention measures such as tamper proof seals, were the most trusted means of identifying authentic and safe foods. Participants across the groups expressed doubts regarding the authenticity of additional cues adopted by manufacturers to signify a products integrity such as certifications and QR codes which were considered to be easily falsified and required additional consumer knowledge to interpret. In addition to product based indicators of authenticity and safety, improved trust and consumer confidence in the Chinese food supply chain was recognised to be contingent upon greater transparency including traceability and improved communication regarding the regulatory measures taken to protect consumer interests in relation to food (Theme 5). Such measures were considered essential in abating Chinese consumer concerns regarding the integrity of the domestic food supply chain and improving the reputation of Chinese food production internationally.

Interpretation by the researchers of the emergent themes led to the following postulations from the qualitative data. The greater the level of perceptual concern regarding food fraud, and the risks this carries, aggravated by low levels of trust in the domestic food supply chain and its actors, the greater the importance placed on assurances of authenticity and the more value consumers place on mechanisms that cumulatively support the demonstration of authenticity. The higher the perceived threat from food fraud and the lower the level of trust in the supply chain to guaranteed the authenticity and safety of foods, the greater the intention of Chinese consumers to purchase foods that offer superior guarantees of authenticity. Chinese consumer attitudes toward the above, may, be subject to regional variations.

### Conceptual model

**Fig 1:**
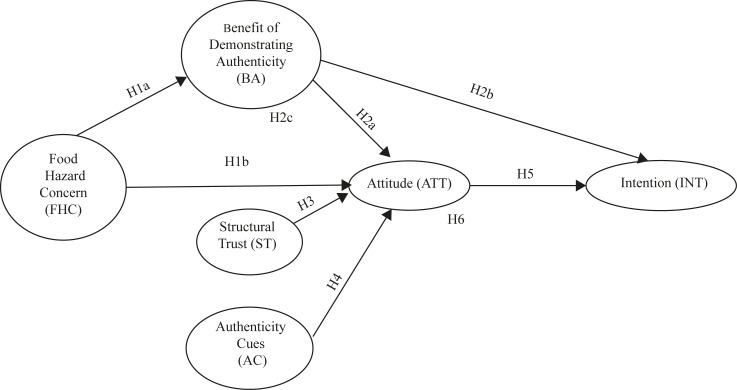
Conceptual model.

Based on insights gained from the focus groups and the literature relating to consumer perceptions of the risks and benefits associated with fraudulent foods, trust in the food system and food risk information provision, a conceptual model was developed (see Fig 1) which drew upon elements of the Theory of Planned Behaviour in order to explore Chinese consumer attitudes towards their consumption environment.

The Theory of Planned Behaviour (TPB) [54, 55]details how behavioural intention can be predicted by attitude, and two additional antecedents; social norms and perceived behavioural control. This model of behavioural intention has been successfully applied in the literature including in relation to several aspects of food choice such as farm animal welfare [56],food safety [57]and personalised nutrition [58].Subsequent studies have also demonstrated that the TPB can be modified to included additional antecedents of behavioural intention [59],such as risks and benefits [58],and factors related to the nature of the behaviour being studied. In the context of this research, additional variables have been added based on the focus group findings (see themes identified in Table 4), specifically to explore the relationship between ‘food hazard concern’, ‘structural trust’, ‘authenticity cues’ and the perceived ‘benefit of demonstrating authenticity’ in the formation of attitude, and the extent to which this predicts intention to purchase authenticated European food products. The association between these different factors are presented in Fig 1.

The emergent themes derived from the analysis of the focus group data (Table 4) and the literature lead to the development of the following seven hypotheses:

**H1**. The greater the concern about food hazards (e.g. food fraud) (FHC) the greater the perceived benefit of demonstrating authenticity (BA) (H1a) and a more positive attitude (ATT) towards authenticated foods (H1b).
**H2.** The greater the perceived benefit of demonstrating authenticity (BA), the more positive attitudes (ATT) towards authenticated foods will be (H2a) and the greater one’s intention (INT) to purchase these (H2b). BA mediates the relationship between FHC and ATT (H2c) (i.e. increased concern about food hazards will result in more positive attitude towards foods demonstrating their authenticity).
**H3.** Greater structural trust (ST) (i.e. trust in food manufacturers, retailers and the government) is negatively associated with positive attitude (ATT) towards authenticated foods.
**H4.** Increased perceived importance associated with authenticity cues (AC) will lead to a more positive attitude (ATT) towards authenticated foods.
**H5.** A positive attitude (ATT) towards ensuring the authenticity of food purchases will increase intention (INT) to purchase authenticated foods.
**H6.** Attitude mediates the effects of food risk concern (FHC), benefit of authenticity (BA), structural trust (ST), and authenticity cues (AC) on intention.
**H7.** The relationships between model paths will be different across locations (i.e. Beijing, Guangzhou, and Chengdu)

#### Sample

Data was collected from the same three cities used in the qualitative study (i.e. Beijing, Guangzhou and Chengdu) to facilitate comparison between stages of the research. Quota sampling was performed in each city, using criteria to ensure that participants were: 1) consumers of IFM, and/or Scotch whisky and/or olive oil, or were intending to purchase one or more of the products in the next three months, 2) had resided in their respective cities for 3 years or more, 4) aged 18–55 years 5) and represented equal percentages of male and female respondents. The final sample consisted of n = 850 study participants who either purchased, or intended to purchase, one of the three product categories. The sample included equal participant representation from the three data collection locations, gender and age categories. Consistent with the focus groups, education and income were used as indicators of socio-economic class and participants represented middle class affluent Chinese consumers; the intended purchasers of imported European foods. The majority of participants were educated to college diploma and degree level or higher, although a higher percentage of participants residing in Beijing were educated to the higher level compared to those residing in Guangzhou and Chengdu. Higher average monthly incomes were recorded for participants residing in Beijing. Only participants residing in Chengdu reported to have average household incomes in the lowest income category, with the majority of participants reporting incomes at this level. No participants residing in Beijing or Guangzhou reported monthly incomes below 1,000 RMB (Table 5).

**Table 5:** Socio-demographic profile of the total sample and individual cities.

Socio-demographic profile	Total Sample	Beijing	Guangzhou	Chengdu	*Sig*.	χ^2^ (df)
	n = 850	n = 284	n = 283	n = 283		
***Gender %***					0.996	0.007 (2)
Male	50	50	49.8	50.2		
Female	50	50	50.2	49.8		
***Age* %**					0.996	0.007 (2)
18–35	50	50	50.2	49.8		
35–55	50	50	49.8	50.2		
***Education*** %					0.000	73.746 (12)
Primary school	0.5	0	0.7	0.7		
Junior high school	5.1	2.1	1.8	11.3		
Senior high school	23.4	18.3	22.6	29.3		
Technical school	6.5	5.3	11.3	2.8		
2–3 years college	26.2	28.9	28.3	21.6		
University	36.5	41.9	35.0	32.5		
Graduate degree or above	1.9	3.5	0.4	1.8		
***Monthly Income %***					0.000	333.549 (6)
RMB 8000–9999	15.2	0	0	45.6		
RMB 10000–14999	46	47.9	48.1	42		
RMB 15000–19999	25.6	36.3	32.2	8.1		
RMB 20000 and above	13.2	15.8	19.4	4.2		
***Purchases %***					0.000	
Intend to purchase only	29.2	25.0	29.3	33.2		56.817 (6)
Infant formula milk only	22.1	16.5	27.2	22.6		
Whisky only	21.2	16.5	27.9	19.1		
Any purchase (Includes olive oil purchasers and people who have purchased two or more from baby milk formula, whisky and olive oil).	27.5	41.9	15.5	25.1		

#### Procedure and measures

An administered survey was used to test the proposed model (see Fig 1). Data collection was again conducted by the social science research agency MB, and all data was collected in August 2015. The questionnaire consisted of closed questions covering eight sections: Section 1 examined consumers’ perceptions related to food and drink hazards in the food system. Section 2 looked at food choice motives. Section 3 contained questions relating to consumers’ trust in the governance of the food system. Section 4 examined consumers’ perceptions of the risk posed by adulterated foods and perceived benefits of demonstrating authenticity. Section 5 explored the importance consumers attached to authenticity cues such as brand and country of origin. Section 6 looked at perception to traceability. Section 7 measured attitudes and intentions. The last section recorded socio-demographic information. The study constructs were measured using multiple items scales that were taken from the literature or devised based on the focus group findings. The English version of the questionnaire is provided see S1 Appendix.

#### Data analysis

All statistical analysis was performed using IBM SPSS 20 [60] and AMOS 20.0 [61].Structural equation modelling (SEM) with maximum likelihood was used to test the conceptual model using Anderson and Gerbing’s [62]two step approach. First, the measurement model was assessed for each city sample through a confirmatory factor analysis (CFM; see S1 Table) and then the full structural model was analysed, which generated overall model fit statistics and significance tests for the model paths across the three for city samples. Several model fit indices were examined including chi-square index, RMSEA (Root Mean Squared Error of Approximation), CFI (Comparative Fit Index) and SRMR (Standardised Root Mean Squared Residual). A good fitting model should have RMSEA OF ≤ 0.05, CFI ≥0.09 and SRMR ≤ 0.08 [63, 64]. Finally, multi-group analysis (MSEM) was performed to assess difference across cities (i.e. Beijing, Guangzhou and Chengdu). This process involves firstly analysing the conceptual model with no constraints (Model 1) across each group (i.e. cities) to test for configural invariance, then analysing the conceptual model with factor loadings constrained (model 2) across each group to test for metric/measurement invariance and finally analysing the conceptual model with regression paths and structural covariance’s constrained in order to test for structural invariance (model 3). In the restricted models (models 2 and 3), model parameters are restricted to be equal for all groups and fit statistics for each restricted SEM model are estimated. If there are no differences between the model fit indices for the unrestricted model (model 1) and the restricted models, the parameters in measurement and structural components of the theoretical model are equivalent (i.e. invariant) across the sub-groups. Evidence of invariance is traditionally determined using the chi-square difference test [65]. If the chi-square statistic (χ^2^) is significant (i.e. ≤ 0.05) then there is evidence that some/all parameters are not invariant across the sub-groups and further tests are required to determine which parameters account for these non-invariant findings. However, chi-square is an excessively stringent test of invariance and recent research suggests that it is more prudent to base invariance decisions on the difference in CFI [65]. A ΔCFI ≤ 0.01, the cut of point proposed by Cheung and Rensvold [66] indicates that the null hypothesis of invariance/equivalence should not be rejected.

### Results

#### Descriptive statistics

Examination of the mean scores for the model constructs across each city reveals a number of similarities (see Table 6). Intention to purchase authenticated products (measured on a 1–10 scale) was found to be similar across the three cities, averaging 8 out of 10 implying that respondents intend to purchase authenticated products. Attitude towards authenticated products (measured on a 1–5 agreement scale) was also positive across the three cities, being slightly lower in Guangzhou (although still averaging 4 out of 5). The importance of authenticity cues was highest in Beijing, although again average scores for the construct across the three cities still 4 (out of 5). Structural trust and the benefits of demonstrating authenticity were again similar across all three cities. However, food hazard concern was different between cities, being lower in Guangzhou compared to Beijing and Chengdu.

**Table 6:** Descriptive statistics and correlation analysis for the model constructs.

**Beijing (n = 284)**						
	FHC (M = 4.18; SD = 1.12	BA (M = 4.47; SD = 0.57)	ST (M = 3.95; SD = 0.87)	AC (M = 4.33; SD = 0.55)	ATT (M = 4.56; SD = 0.60)	INT (M = 8.73; SD = 1.64
FHC	1.00					
BA	0.20**	1.00				
ST	0.86	0.37**	1.00			
AC	0.21**	0.55**	0.49**	1.00		
ATT	0.21**	0.34**	0.17**	0.34**	1.00	
INT	0.241**	0.31**	0.19**	0.31**	0.42**	1.00
**Guangzhou (n = 283)**						
	FHC (M = 3.91; SD = 0.97)	BA (M = 4.10; SD = 0.51)	ST (M = 3.86; SD = 0.56)	AC (M = 3.94; SD = 0.42)	ATT (M = 4.17; SD = 0.50)	INT (M = 8.22; SD = 1.11)
FHC	1.00					
BA	0.39**	1.00				
ST	0.24**	0.34**	1.00			
AC	0.22**	0.42**	0.39**	1.00		
ATT	0.03	0.26**	0.06	0.37**	1.00	
INT	0.18**	0.30**	0.16**	0.12*	0.01	1.00
**Chengdu (n = 283)**						
	FHC (M = 4.44; SD = 0.61)	BA (M = 4.4; SD = 0.51)	ST (M = 3.75; SD = 0.81)	AC (M = 4.01; SD = 0.49)	ATT (M = 4.50; SD = 0.59)	INT (M = 8.15; SD = 1.54)
FHC	1.00					
BA	0.29**	1.00				
ST	0.17**	0.31**	1.00			
AC	0.27**	0.47**	0.43**	1.00		
ATT	0.33**	0.49**	0.17**	0.38**	1.00	
INT	0.21**	0.39**	0.08	0.20**	0.37**	1.00

*p ≤ 0.05

**p≤ 0.01

M = mean and SD = standard deviation. FCH = Food Hazard Concern, BA = Benefit of Authenticity, ST = Structural Trust, AC = Authenticity cues, ATT = Attitude, ITT = Intention.

When examining the correlations between model constructs, the relationships differ considerably between cities. As shown in Table 6, correlations range from 0.17 to 0.55 in the Beijing sample, from 0.12 to 0.42 in the Guangzhou sample, and from 0.17 to 0.47 in the Chengdu sample. In particular, the correlation between structural trust and food hazard concern is much greater in Beijing compared to Chengdu and Guangzhou, and the correlation between intention to purchase and attitude is much lower in Guangzhou compared to the other two cities.

#### Evaluation of the measurement model

Evaluation of the six-factor measurement model by means of CFA shows that it provided acceptable fit across the samples (CFI = 0.95_Beijing_, 0.92_Guangzhou_, 0.90_Chengdu_; RMSEA = 0.05 across the three samples; SRMR = 0.05_Beijing_, 0.07_Guangzhou_, 0.06_Chengdu_). All measured items significantly loaded on their constructs (p <0.001). Cronbach alpha scores for each construct are greater than the generally accepted limit of 0.7 except for ATT (α = 0.58 _Beijing_; 0.58 _Guangzhou_; 0.68 _Chengdu_). Kline [67] notes that for psychological variables, values below 0.7 can be expected. Nunally [68] as cited in [69], suggested that a reliability score from 0.5 to 0.6 is the minimum acceptable level. Mean and standard deviations per construct and correlations between constructs are provided in Table 6. Correlations range from 0.17 to 0.55 in the Beijing sample; from 0.12 to 0.42 in the Guangzhou sample; from 0.17 to 0.47 in the Chengdu sample.

#### Evaluation of the structural model

Assessment of normality across the samples shows that the data are non-normally distributed as univariate skewness values ranged from -0.02 to -2.86 and kurtosis values ranged from -0.02 to 9.53, while Mardia’s normalised coefficient of multivariate kurtosis is greater than 5.00 in the three samples. According to Bentler [70],values above 5.00 indicate that the data are not multivariate normal. Consequently, a bootstrap procedure (2000 replications) was used to test the model fit and the bias-corrected regression coefficients are reported for the structural model. The fit indices (CFI, SRMR, and RMSEA) indicate that the model was an adequate to good fit for the data across the three cities (Table 6). In the Beijing sample, the model explained 32% (*R*^2attitude^ = 0.318) variance in attitudes towards ensuring food and drink products that have been traced for authenticity and 35% (*R*^2Intention^ = 0.349) variance in intentions to purchase food products that have been traced for authenticity. In the Guangzhou sample the model explained 52% (*R*^2attitude^ = 0.52) variance in attitudes and 22% (*R*^2Intention^ = 0.22) variance in intentions. In the Chengdu sample the model explained 51% (*R*^2attitude^ = 0.51) variance in attitudes and 28% (*R*^2Intention^ = 0.38) variance in intentions.

**Fig 2:**
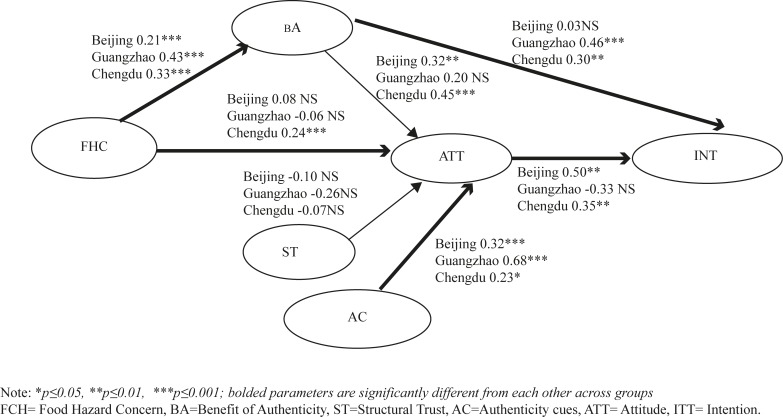
Structural paths and multi-group analysis.

Table 7 and Fig 2 show results for hypothesised paths in each sample. Significant results were found for a number of parameter estimates supporting the hypotheses, apart from H1b, H2a, H3 and H5 for Guangzhou, H2b and H3 for Beijing and H3 for Chengdu. This implies that structural trust (ST) is not a significant influence on attitude to purchase authenticated products for consumers in these cities. Results indicate that FHC is a concern across each of the three cities although significant differences exist between them in terms of how this is manifested (e.g. in relation to H1a and H1b). For Beijing and Chengdu FHC manifests itself through ATT and BA, or solely through BA in the case of Guangzhou. Authenticity cues were an important influence on attitude across the three cities, especially for Guangzhou.

**Table 7:** Structural paths, multi-group analysis and structural invariance.

Hypotheses		Beijing	Guangzhou	Chengdu	Δ χ^2^	Δ d.f.	p
	*Direct Effects*						
**H1a**	FHC—BA	0.21***	0.43***	0.33***	9.426	2	<0.01
**H1b**	FHC—ATT	**0.08**	**-0.06**	0.24***	12.522	2	<0.01
**H2a**	BA—ATT	0.33**	0.22	0.46***	4.706	2	>0.05
**H2b**	BA—INT	**0.03**	0.46***	0.30**	7.995	2	<0.05
**H3**	ST—ATT	-0.10	-0.26	-0.07	2.784	2	>0.05
**H4**	AC—ATT	0.32**	0.68***	0.23*	6.668	2	<0.05
**H5**	ATT—INT	0.50***	**-0.33**	0.35**	7.022	2	<0.05
	*Indirect Effects*						
**H2c**	FHC–BA—ATT	0.07*	0.10	0.15***			
**H6**	FHC–ATT—INT	0.08	0.19*	0.24***			
**H6**	BA–ATT—INT	0.16*	-0.07	0.16**			
**H6**	ST–ATT- INT	-0.05	0.09	-0.03			
**H6**	AC–ATT—INT	0.16**	- 0.23	0.08*			

Notes

*p≤0.05

**≤ 0.01

***p ≤ 0.001

Bolded parameters are significantly different from each other across groups. FCH = Food Hazard Concern, BA = Benefit of Authenticity, ST = Structural Trust, AC = Authenticity cues, ATT = Attitude, ITT = Intention.

#### Multi-group analysis

The qualitative research highlighted differences in attitudes towards food fraud, geographically among sample cites and between products. Consequently, multi-group structural equation (MSEM) modelling was conducted to examine the moderating influence of geographical location of the conceptual mode, therefore testing H7. As shown in Table 8, based on the ΔCFI criteria, configural invariance (model 1) and metric/measurable variance (model 2) were observed but structural invariance (model 3) was not as ΔCFI was > 0.01. Having identified evidence of structural non-invariance for location (i.e. the structural regression paths and covariance paths are not the same for Beijing, Guangzhou, Chengdu), the next step was to determine which structural regression paths are non-invariant in order to fully test the final study hypothesis (H7), this entailed constraining each structural regression path separately and examining change in chi-square (Δχ^2^) when compared against the baseline model i.e. all the structural paths are free to vary across the groups. The results show significant non-invariance for the paths FHC → BA (Δχ^2^ (2) = 9.426, p <0.01), FHC → ATT (Δχ^2^ (2) = 12.522, p <0.01), BA → INT (Δ*x*^2^(2) = 7.995, p <0.05), AC → ATT (Δ*x*^2^(2) = 6.668, p <0.05), AC → INT (Δχ^2^ (2) = 7.022, p <0.05). Paths significantly different among samples are reported in bold in Fig 2 and Table 7.

**Table 8:** Goodness of fit indices for tests of multi-group invariance.

Model	N	χ^2^	d.f.	P	RMSEA	SRMR	CFI	ΔCFI
**Location**								
Beijing	284	736.140	447	<0.001	0.048	0.493	0.950	
Guangzhou	283	706.631	447	<0.001	0.045	0.674	0.921	
Chengdu	283	828.773	447	<0.001	0.055	0.635	0.903	
Model 1 (C.I.)	850	2266.544	1341	<0.001			0.928	
Model 2 (M.I)	850	2408.454	1393	<0.001			0.921	0.007
Model 3 (S.I.)	850	2655.661	1429	<0.001			0.905	0.023

Note: C.I. = Configural invariance, M.I. = Metric Invariance, S.I. Structural invariance.

### Discussion

This research adopted a mixed method research design, combining quantitative and qualitative approaches. The two-stage approach aimed to provide contextual insights into consumer attitudes towards, and experiences of food fraud. When combined with theoretical insights from the literature, the relationship between attitudes and intention to purchase authenticated European food products could be examined and relevant hypotheses tested. The research highlighted deep-rooted concerns amongst Chinese consumers regarding the integrity and safety standards of the domestic food supply chain. Consistent with the literature, consumer concerns were noted to have been perpetuated in the wake of multiple food scandals reported in the Chinese media, the most notable being the 2008 melamine in the IMF scandal ([20, 71–73]).

Findings from the qualitative investigation indicate Chinese consumers to be worried about the risks posed by fraud to the domestic food supply chain and consider this to represent a significant risk to the safety of food that is manufactured in China. The survey results indicate that Chinese consumers have positive attitudes towards food that can be demonstrated to be authentic, and express high levels of intention to purchase authenticated food, whilst the qualitative findings indicate that this level of risk concern was not found to extend to other countries. The Chinese domestic food supply chain was regarded to be inferior to that in other countries, and imported food products were perceived to be superior to products that were domestically produced [72, 74][. Findings from the qualitative research showed Chinese consumers recognised the strict production and regulatory standards that are applied to European food and considered food from Europe to be more reliable in terms of authenticity, safety and quality than domestic comparatives. Imported foods are associated with quality and safety guarantees is a finding that is consistent with previous research (see [72, 74]).

Although the majority of incidents of food adulteration do not cause public health concerns, there are notable exceptions where the contaminants can result in serious risks to consumer health [75]. Chinese consumers participating in this research were unable to disassociate incidents of economically motivated adulteration from food safety risks that, by implication, present potential health risks. The link between authenticity and food safety concerns was a recurrent theme in the focus group discussions, which was reinforced by the well-documented melamine incident in China and the impact that this had upon the health of vulnerable consumers. The potential unknown and/or cumulative long-term health effects of adulterated food was of high concern and consumers were unable to appreciate that some fraudulent practices did not represent a health risk. Findings from the quantitative research further supported this, showing food fraud to be a hazard concern across the three sample cities. Chinese consumers were shown to be most worried about food that had been intentionally adulterated, counterfeited or mis-described (see [20]). The risk posed by food fraud manifested itself in Chinese consumer’s attitude towards authenticated food products and the importance of authenticity cues as an aid to the identification of authentic and safe food products. Regional disparities regarding the level of perceived food hazard concern and intention to purchase authenticated food were identified. Consumers in Chengdu were shown to hold the greatest level of concern compared to consumers residing in Beijing and Guangzhou. Qualitative evidence highlighted the perceived difference in regulatory standards between tier one and two cities, with regulatory efforts purported to be concentrated in the former, which supports pervious research findings [76]. Consequently, consumers residing in lower tier cities are likely to place greater emphasis on demonstrating authenticity. It is noteworthy that consumers residing in Guangzhou had the lowest levels of food risk concern and intention to purchase authenticated food products but placed the highest importance on the use of authenticity cues to support the identification of authentic food products. A possible explanation for this difference in perception of risk emerged from the qualitative analysis. Guangzhou is geographically located close to the territories of Macau and Hong Kong, which were considered by consumers to operate more stringent food safety regulations and enforcement than mainland China. Travel to these states represented an alternative purchasing mechanism that allowed consumers to make purchases in markets that they considered to be more reliable and where the authenticity of food products could be better guaranteed. Food manufacturers, exporters and policy makers are encouraged to recognise regional difference in risk perceptions and the variation in the importance of authenticity cues to consumers when identifying authentic food. Additional reassurances could be provided to consumers by tailoring communication messages, and providing additional reassurances through iconic and indexical cues on products destined for sale in areas where the perception of risk posed by food fraud is greater.

Low levels of structural trust in actors involved in the Chinese domestic food supply chain, and its governance was a prominent theme to emerge from the qualitative research. However, the survey did not reveal structural trust to be an important predictor of attitude and intention to purchase authenticated food. The resignation of Chinese consumers to the risks associated with food that is domestically produced reported within the qualitative research provides a possible explanation as to why this was not found to be significant. In the absence of structural trust and in response to the dissonance arising from food fraud consumers had developed a range of ‘risk-relieving’ strategies which could be considered coping mechanisms, as a consequence of the perceived prevalence of inauthentic or fraudulent food in the domestic market. Consumers had developed a range of pre- and post-purchase and consumption strategies, that in the absence of trust in the domestic market supported perceptions of the integrity of the food they purchased and consumed. The strategies included; pre-purchase information searching, carefully selected acquisition sources (including travel to neighbouring states and informal import networks through friends and family living abroad), the use of tangible iconic and indexical cues provided by product manufacturers as means of communicating the authenticity of products and an array of domestically situated practices [77]. The importance of authenticity cues had a positive direct effect on consumers’ attitudes towards purchasing authenticated food products, and an indirect effect *via* attitude on intention to purchase. Across all three-sample cities consumers were shown to rely upon indexical and iconic cues of authenticity. Difference in the level of importance attached to these cues were identified, with consumers in Beijing and Chengdu placing less importance on authenticity cues than those living in Guangzhou. The findings of the qualitative work highlighted consumers residing in Beijing and Chengdu to be less trusting of tangible product cues. Consumers in these cities were of the belief that indexical and iconic cues of authenticity provided by food manufacturers could be easily falsified by fraudsters. The alternative purchasing channels available to consumers in Guangzhou through travel to neighbouring states, provided authenticity and safety assurances and gave greater confidence in the reliability of authenticity cues. Qualitative investigation of which cues of authenticity were preferred and used by consumers to help identify authentic products revealed preference for traditional cues such as brand, price and packaging to be the most trusted indicators of authenticity. Examination of a product’s packaging was important in verifying authenticity with consumers reporting checking product seals for signs of tampering and the quality of the printing to avoid suspected counterfeits. Although consumers were aware that food manufacturers had developed more sophisticated ways of verifying products authenticity and evidencing traceability through for example, the use of quick response codes (QR), consensus across the focus groups suggested that consumers regarded these to be cumbersome, adding additional time to the purchasing process and consequently were infrequently used.

The same level of structural distrust was shown not to extend to foreign markets, with Chinese consumers perceiving imported food products to offer greater levels of consumer protection. Across the focus groups consumers reported that they sought out imported products as an additional strategy for ensuring the integrity of products. From the perspective of European food manufacturers, therefore, it is important to highlight the country of origin within Europe in order to differentiate their products from domestically produced equivalents. It is also notable that, in lieu of trust in governance structures and food manufacturers, the qualitative research indicated that Chinese consumers sought reassurances from their immediate social networks (i.e. family and friends) as formative sources of information with regards to authentic products and trusted retailers (see also [20]).

The structural equation model developed may be applicable in relation to predicting consumer responses to food inauthenticity in other contexts, although further cross–cultural testing of the model is required. In general, the initial hypothesis regarding the relationship between consumer concern regarding food fraud, structural trust, attitude and behavioural intention to purchase authentic products was supported for Chinese participants. Given that the hypotheses were generated using a combination of qualitative research (using Chinese participants) and the broader literature, there is no reason to doubt their generalisability, although there will need to be a broader empirical test to assess the cross-cultural validity of the scales in the future. However, the scale is potentially a useful tool in assessing the drivers of consumer reactions to food fraud which may be utilised as a basis for future research activities. It is also of note that the attitudinal variables considered in the model have similarities with results identified within other areas of food related application. The positive relationship between attitude and behavioural intention has been confirmed in many other studies (for example, see *inter alia* [39, 78, 79]). Lower structural trust was not associated with positive attitudes towards authenticity, suggesting that as structural trust decreases, consumers are more likely to require greater reassurance regarding the efficacy and enforcement of regulations. This has been reported in other food related areas, such as food risk perception and risk management, or adoption of nutrigenomics (for example, see [57, 58, 80, 81]). The research therefore lends credence to attempts at building more generic theories of the relationships between risk and benefit perception and structural trust (as proposed, for example, by [82, 83].

The findings of this research are not without their limitations. Given the nature of the intended target market of the European products selected as the focus of this research, the sample included only middle class affluent Chinese consumers. This, therefore limits the potential to generalise the findings of this research to the Chinese population as a whole. Whilst urban incomes continue to grow rapidly and affluent middle class Chinese consumers represent a growing market segment particularly for high value European food products it is recommended that future research includes a greater socio-economic range of consumers.

### Conclusions

Chinese consumers are concerned about the pervasive nature of fraudulent activity within the domestic food supply chain and consider this to represent a food safety risk. Despite considerable efforts to improve the integrity of the domestic food supply chain in China, Chinese consumers have low levels of trust in food that is domestically produced and perceive food that has been produced in China to be of inferior quality and safety to food produced in other countries. Despite the lack of trust Chinese consumers have in the domestic food supply chain, this was not found to influence consumer’s attitude or intention to purchase authenticated food—rather Chinese consumers have developed a range of ‘risk-relieving’ strategies to personally alleviate their food risk concerns. The ability to individually manage risk moderated the lack of trust in the food chain. Indexical and iconic cues of authenticity provided by food manufacturers and regulators may mitigate perceived health risks and provide assurances of a product’s authenticity. Therefore, when considering how to differentiate product offerings, European food manufacturers operating in the Chinese market should pay consideration to additional mechanisms used by consumers to support the identification of authentic products, including where and from whom consumers obtain information regarding trustworthy products and how products are sourced. Improved transparency in communications from policy makers and industry in actions taken to protect consumers would further support Chinese consumers to identify authentic food products and more importantly act to re-build trust in the domestic food supply chain. Communications should serve to reassure consumers about the safety of products, in addition to the authenticity cues provided on labels. This will also improve trust in regulations, those responsible for standards, and the food industry and reduce consumer’s reliance upon personal risk mitigation strategies.

## Supporting information

S1 Appendix Blank survey instrument. (DOCX)

S1 Table Confirmatory factor analysis. Item list and loadings per construct.
(DOCX)

S1 Dataset (XLSX)
