# Supplementary material for: Food fraud and the perceived integrity of European food imports into China
Source: PLoS One. 2018 May 23;13(5):e0195817. doi: 10.1371/journal.pone.0195817 (PMC5965827; doi:10.1371/journal.pone.0195817)
Supplement: S1 Appendix — (DOCX) [file pone.0195817.s001.docx]

**S1 Appendix. Blank survey instrument.**

*SECTION ONE: RISK PERCEPTIONS RELATING TO FOOD AND DRINK*

- 1. **To what extent do you agree/disagree that you are worried about each of the following:**

Please indicate your response on a 1-5 scale where 1=Strongly disagree, 2= disagree, 3=Neither agree nor disagree, 4=Agree and 5= Strongly agree.

| **Hazard** | **Strongly disagree** | **Disagree** | **Neither agree nor disagree** | **Agree** | **Strongly agree** |
| --- | --- | --- | --- | --- | --- |
|  | **1** | **2** | **3** | **4** | **5** |
| Counterfeit food & drink |  |  |  |  |  |
| Inferior quality food & drink |  |  |  |  |  |
| Food& drink containing residues of pesticides or veterinary drugs |  |  |  |  |  |
| Deteriorated food & drink (for example, mould and bacteria) |  |  |  |  |  |
| Nutritionally imbalanced food & drink |  |  |  |  |  |
| Food & drink containing additives |  |  |  |  |  |
| Genetically Modified food & drink |  |  |  |  |  |
| Food & drink that has been miss-described |  |  |  |  |  |
| Adulterated food & drink (for example: tainted food & drink, food & drink that has been diluted or had chemicals added) |  |  |  |  |  |

- 1. **To what extent do you agree/disagree that the following food hazards pose a serious food and drink risk?**

Please indicate your response on a 1-5 scale where 1=Strongly disagree, 2= disagree, 3=Neither agree nor disagree, 4=Agree and 5= Strongly agree.

| **Hazard** | **Strongly disagree** | **Disagree** | **Neither agree nor disagree** | **Agree** | **Strongly agree** |
| --- | --- | --- | --- | --- | --- |
|  | **1** | **2** | **3** | **4** | **5** |
| Counterfeit food & drink |  |  |  |  |  |
| Inferior quality food & drink |  |  |  |  |  |
| Food & drink containing residues of pesticides or veterinary drugs |  |  |  |  |  |
| Deteriorated food & drink (for example, mould and bacteria) |  |  |  |  |  |
| Nutritionally imbalanced food & drink |  |  |  |  |  |
| Food & drink containing additives |  |  |  |  |  |
| Genetically Modified food & drink |  |  |  |  |  |
| Adulterated food & drink (for example: tainted food & drink, food & drink that has been diluted or had chemicals added) |  |  |  |  |  |

*SECTION TWO: FOOD AND DRINK CHOICES*

**2.1.** **I would now like you to think about the factors that influence your food and drink choices. I will read a list of factors, please tell me the extent to which you agree/disagree that each of the following are important considerations when choosing food & drink products to buy and eat.**

Please indicate your response on a scale of 1 to 5 where 1= strongly disagree, 2= Disagree, 3=Neither agree nor disagree, 4= agree and 5= strongly agree.

| **Factors influencing the food & drink you choose** | **Strongly disagree** | **Disagree** | **Neither agree nor disagree** | **Agree** | **Strongly agree** |
| --- | --- | --- | --- | --- | --- |
|  | **1** | **2** | **3** | **4** | **5** |
| Contains a lot of vitamins and minerals |  |  |  |  |  |
| Keeps you healthy |  |  |  |  |  |
| Is nutritious |  |  |  |  |  |
| Is high in protein |  |  |  |  |  |
| Is good for you |  |  |  |  |  |
| Is good for your skin/teeth/hair/nails etc. |  |  |  |  |  |
| Is high in fibre and roughage |  |  |  |  |  |
| Helps you to cope with stress |  |  |  |  |  |
| Helps you to cope with life |  |  |  |  |  |
| Helps you to relax |  |  |  |  |  |
| Keeps you awake/alert |  |  |  |  |  |
| Cheers you up |  |  |  |  |  |
| Makes you feel good |  |  |  |  |  |
| Is easy to prepare |  |  |  |  |  |
| Can be cooked very simply |  |  |  |  |  |
| Takes no time to prepare |  |  |  |  |  |
| Can be bought in shops close to where you live or work |  |  |  |  |  |
| Is easily available in shops and supermarkets |  |  |  |  |  |
| Smells nice |  |  |  |  |  |
| Looks nice |  |  |  |  |  |
| Has a pleasant texture |  |  |  |  |  |
| Tastes good |  |  |  |  |  |
| Contains no additives |  |  |  |  |  |
| Contains natural ingredients |  |  |  |  |  |
| Contains no artificial ingredients |  |  |  |  |  |
| Is not expensive |  |  |  |  |  |
| Is cheap |  |  |  |  |  |
| Is good value for money |  |  |  |  |  |
| Is low in calories |  |  |  |  |  |
| Helps you control your weight |  |  |  |  |  |
| Is low in fat |  |  |  |  |  |
| Is what you normally eat |  |  |  |  |  |
| Is well-known |  |  |  |  |  |
| Is like the food you ate when you were a child. |  |  |  |  |  |
| Comes from countries you approve of politically |  |  |  |  |  |
| Has the country of origin clearly marked |  |  |  |  |  |
| Is packaged in an environmentally friendly way |  |  |  |  |  |

*SECTION THREE: RESPONSIBILITYAND TRUST*

We are now going to ask you to think about who should be responsible for protecting consumers and ensuring that they have safe and authentic food and drink.

<DEFINITION OF AUTHENTIC> By ‘authentic food and drink’ we mean that the food and drink that has not been tampered with in any way and it is what it says it is.

**3.1 To begin, I will read out a list of statements about food and drink protection regulations in China. Please tell me to what extent you agree/disagree with each of the statements:**

Please indicate your response on a 1 to 5 scale where 1= Strongly Disagree, 2= Disagree , 3= Neither Agree nor Disagree, 4= Agree, 5= Strongly Agree .

| **Food and drink protection in China** | **Strongly Disagree** | **Disagree** | **Neither Agree nor Disagree** | **Neither** | **Strongly Agree** |
| --- | --- | --- | --- | --- | --- |
|  | **1** | **2** | **3** | **4** | **5** |
| Current regulations in China are adequate to protect consumers from the potential risks of food & drink adulteration |  |  |  |  |  |
| Current enforcement of regulations are adequate to protect consumers from the potential risks of food & drink adulteration. |  |  |  |  |  |
| There are adequate procedures in place to ensure that everyone benefits from safe food & drink that has not been adulterated. |  |  |  |  |  |

**3.2** **Which of the** **following organisations or individuals should be responsible for ensuring that consumers have safe and authentic food and drink?**

Please indicate your response using a 1 to 5 where 1= Strongly Disagree, 2=Disagree, 3=Neither Agree nor Disagree, 4=Agree and 5=Strongly Agree, the extent to agree or disagree.

| **Organisations /Individuals** | **Strongly Disagree** | **Disagree** | **Neither agree nor disagree** | **Agree** | **Strongly Agree** |
| --- | --- | --- | --- | --- | --- |
|  | **1** | **2** | **3** | **4** | **5** |
| Food & drink retailers |  |  |  |  |  |
| Food & drink manufacturers |  |  |  |  |  |
| Consumer organizations |  |  |  |  |  |
| Medical doctors |  |  |  |  |  |
| Dieticians/nutritionists |  |  |  |  |  |
| Friends and family |  |  |  |  |  |
| The media |  |  |  |  |  |
| Government |  |  |  |  |  |
| Other |  |  |  |  |  |
| Other please specify: |  | | | | |

**3.3** **I will read a list of organisations and individuals and I would like you to tell me the extent to which you agree/disagree that they can be trusted to protect the consumer from adulterated and unsafe food and drink?**

<Definition of Adulterated Food and Drink> by ‘adulterated food and drink’ we mean that the food or drink product has been tampered with in some way and it is not what it says it is.

Please rate your response on a 1 to 5 scale where 1= strongly disagree, 2= Disagree, 3= Neither agree nor disagree, 4= Agree, 5= Strongly agree.

| **Organisations/ Individuals** | **Strongly disagree** | **Disagree** | **Neither agree nor disagree** | **Agree** | **Strongly agree** |
| --- | --- | --- | --- | --- | --- |
|  | **1** | **2** | **3** | **4** | **5** |
| Food retailers |  |  |  |  |  |
| Food manufacturers |  |  |  |  |  |
| Consumer organizations |  |  |  |  |  |
| Medical doctors |  |  |  |  |  |
| Dieticians/nutritionists |  |  |  |  |  |
| Friends and family |  |  |  |  |  |
| The media |  |  |  |  |  |
| Government |  |  |  |  |  |
| Other |  |  |  |  |  |
| Other please specify: |  | | | | |

**3.4. I will read a list of organisations and individuals and I would like you to tell me the extent to which you agree/disagree that they can be trusted to provide consumers with accurate sources of information about the authenticity of food and drink?**

Please rate your response on a 1 to 5 scale where 1= Strongly disagree, 2= Disagree, 3= Neither agree nor disagree, 4=Agree, 5= Strongly Agree.

| **Information source** | **Strongly disagree** | **Disagree** | **Neither agree nor disagree** | **Agree** | **Strongly agree** |
| --- | --- | --- | --- | --- | --- |
|  | **1** | **2** | **3** | **4** | **5** |
| Food retailers |  |  |  |  |  |
| Food manufacturers |  |  |  |  |  |
| Consumer organizations |  |  |  |  |  |
| Medical doctors |  |  |  |  |  |
| Dieticians/nutritionists |  |  |  |  |  |
| Friends and family |  |  |  |  |  |
| The media |  |  |  |  |  |
| Government |  |  |  |  |  |
| Other |  |  |  |  |  |
| Other please specify: |  | | | | |

*SECTION FOUR: RISKS AND BENEFITS*

**4.1. I will read out a list of potential risks posed by adulterated food and drink products. Please indicate the extent to which you agree/disagree that the adulterated food and drink poses a risk to:**

Please indicate your response using a 1 to 5 scale where 1=Strongly Disagree, 2=Disagree, 3=Nether agree nor Disagree, 4=Agree and 5=Strongly Agree**.**

| **Adulterated food and drink poses a risk to:** | **Strongly Disagree** | **Disagree** | **Neither Agree nor Disagree** | **Agree** | **Strongly Agree** |
| --- | --- | --- | --- | --- | --- |
|  | **1** | **2** | **3** | **4** | **5** |
| Consumer health |  |  |  |  |  |
| You personally |  |  |  |  |  |
| Your family |  |  |  |  |  |
| Food & drink quality |  |  |  |  |  |
| The environment |  |  |  |  |  |
| The economy |  |  |  |  |  |
| The taste of food & drink |  |  |  |  |  |
| Food & drink safety |  |  |  |  |  |
| The nutritional quality of food & drink |  |  |  |  |  |
| The reputation of China |  |  |  |  |  |
| Your confidence in the food supply chain |  |  |  |  |  |
| Your confidence in food regulators |  |  |  |  |  |
| Your confidence in the government |  |  |  |  |  |
| Consumer trust in the food they buy |  |  |  |  |  |

**4.2. I will read out a list of potential benefits demonstrating the authenticity of food and drink products. To what extent do you agree/ disagree that demonstrating the authenticity of food & drink will be of benefit to:**

Please indicate your position on a scale of 1= Strongly Agree, 2= Agree, 3= Neither Agree nor Disagree, 4= Disagree, 5= Strongly Disagree.

| **Benefit of demonstrating authenticity** | **Strongly Disagree** | **Disagree** | **Neither Agree nor Disagree** | **Agree** | **Strongly Agree** |
| --- | --- | --- | --- | --- | --- |
|  | **1** | **2** | **3** | **4** | **5** |
| Improve consumer health |  |  |  |  |  |
| Improve food & drink safety |  |  |  |  |  |
| Improve food & drink quality |  |  |  |  |  |
| Be beneficial to you |  |  |  |  |  |
| Be beneficial to your family |  |  |  |  |  |
| Improve consumer trust |  |  |  |  |  |
| Be beneficial to the environment |  |  |  |  |  |
| Be beneficial to the Chinese economy |  |  |  |  |  |
| Increase the reliability of food & drink products |  |  |  |  |  |
| Improve the reputation of China |  |  |  |  |  |
| Improve trust in the food chain |  |  |  |  |  |
| Improve trust in food regulators |  |  |  |  |  |
| Improve trust in food & drink products |  |  |  |  |  |
| Improve trust in Chinese food & drink manufacturing |  |  |  |  |  |
| Improve trust in government |  |  |  |  |  |

*SECTION FIVE: AUTHENTICITY CUES*

**5.1.** **Please tell me which of the following you would consider to be important when making judgements about the authenticity of a food and drink products?**

Please rate your response on a scale from 1 to 5 where 1=Strongly Disagree, 2=Disagree, 3=Neither Agree nor Disagree, 4=Agree and 5=Strongly Agree.

| **Authenticity Cue** | **Strongly disagree** | **Disagree** | **Neither agree nor disagree** | **Agree** | **Strongly Agree** |
| --- | --- | --- | --- | --- | --- |
|  | **1** | **2** | **3** | **4** | **5** |
| Country of origin |  |  |  |  |  |
| A product that is produced and packaged in Europe |  |  |  |  |  |
| A product that is produced and packaged in China |  |  |  |  |  |
| The brand |  |  |  |  |  |
| The nutritional information |  |  |  |  |  |
| The price |  |  |  |  |  |
| The packaging |  |  |  |  |  |
| The retailer |  |  |  |  |  |
| The product has a tamper proof seal |  |  |  |  |  |
| The product has a certificate of authenticity |  |  |  |  |  |
| The barcode |  |  |  |  |  |
| Other |  |  |  |  |  |
| Other Please specify: |  | | | | |

*SECTION SIX: TRACEABILITY*

**6.1.** **To what extend do you agree /disagree that it is important to know the origin of the following food or drink products?**

Please rate your response on a 1 to 5 scale where 1=Strongly Disagree, 2= Disagree, 3=Neither Agree nor Disagree, 4=Agree and 5=Strongly Agree.

| **Food type** | **Strongly Disagree** | **Disagree** | **Neither agree nor disagree** | **Agree** | **Strongly Agree** |
| --- | --- | --- | --- | --- | --- |
|  | **1** | **2** | **3** | **4** | **5** |
| All food & drink products |  |  |  |  |  |
| Food & drinks I buy in restaurants |  |  |  |  |  |
| Food & drinks I consume at home |  |  |  |  |  |
| Green food & drink products |  |  |  |  |  |
| Luxury food & drink products |  |  |  |  |  |
| Imported food & drink products |  |  |  |  |  |
| Food & drinks I give as gifts |  |  |  |  |  |
| Food & drink that I personally consume |  |  |  |  |  |
| Food & drink that my family consume |  |  |  |  |  |
| Other |  |  |  |  |  |
| Other Please specify: |  | | | | |

*SECTION SEVEN: PURCHASE INTENTIONS*

- 1. **I am going to ask you some questions about your purchasing attitudes and intentions. Please tell me the extent to which you agree/disagree with the following statements:**

Please rate your response on a 1 to 5 scale where 1=Strongly Disagree, 2= Disagree, 3=Neither Agree nor Disagree, 4=Agree and 5=Strongly Agree.

| **For me ensuring the authenticity of the food & drink that I buy and eat is:** | **Strongly Disagree** | **Disagree** | **Neither Agree nor Disagree** | **Agree** | **Strongly Agree** |
| --- | --- | --- | --- | --- | --- |
|  | **1** | **2** | **3** | **4** | **5** |
| Beneficial |  |  |  |  |  |
| Unimportant |  |  |  |  |  |
| Essential |  |  |  |  |  |

- 1. **I am going to read three statements and I would like you to tell me on a 1-10 scale the extent to which you agree/disagree. 1=Strongly disagree and 10=strongly agree**

| Statement | **Strongly Disagree Strongly Agree** | | | | | | | | | |
| --- | --- | --- | --- | --- | --- | --- | --- | --- | --- | --- |
|  | **1** | **2** | **3** | **4** | **5** | **6** | **7** | **8** | **9** | **10** |
| I intend to purchase food and drinks which have been traced for authenticity |  |  |  |  |  |  |  |  |  |  |
| I plan to purchase food and drinks which have been traced for authenticity |  |  |  |  |  |  |  |  |  |  |
| I will try to purchase authentic food and drinks which have been traced for authenticity |  |  |  |  |  |  |  |  |  |  |

We would know like to ask you about food and drink products that you buy.

**7.3** Do **you buy any of the following products?:**

|  | **Yes** | **No** |
| --- | --- | --- |
| **Infant formula milk** |  |  |
| **Whisky** |  |  |
| **Olive oil** |  |  |

*Q 7.4 to be asked depending upon participant response to Q7.1.*

- 1. **How much more would you be willing to pay for a) infant formula milk b) olive oil c) that is authentic and has not been adulterated?**

| 1. **Infant formula milk** | 0 ¥ XX ¥ |
| --- | --- |
| 1. **Olive oil** | 0 ¥ XX ¥ |
| 1. **Whisky** | 0 ¥ XX ¥ |

*SECTION EIGHT: DEMOGRAPHICS*

**9.1 Gender**

| Male |  |
| --- | --- |
| Female |  |

**9.2 How old are you?**

| Below 15 | 1 |
| --- | --- |
| 15-17 | 2 |
| 18-24 years | 3 |
| 25-30 years | 4 |
| 31-35years | 5 |
| 46-40 years | 6 |
| 41-45 years | 7 |
| Above 45 | 8 |

**9.3 Which of the following best describe your education level**

| No formal education | 1 |
| --- | --- |
| Primary school | 2 |
| Junior high school | 3 |
| Senior high school | 4 |
| Technical school | 5 |
| 2-3 years college | 6 |
| University | 7 |
| Graduate degree of above | 8 |

**9.4 Which of the following best describes your parent’s education level?**

| No formal education | 1 |
| --- | --- |
| Primary school | 2 |
| Junior high school | 3 |
| Senior high school | 4 |
| Technical school | 5 |
| 2-3 years college | 6 |
| University | 7 |
| Graduate degree of above | 8 |

**9.4 What is your monthly household income?**

| Below 3000 yuan | 01 |
| --- | --- |
| 3000 - 4999 | 08 |
| 5000 - 7999 | 09 |
| 8000 - 9999 | 12 |
| 10000 - 14999 | 13 |
| 15000 - 19999 | 14 |
| 20000 and above | 15 |
| Refused (don’t read out) | 79 |

**9.5 On average, what is your monthly spend on grocery shopping?**

**Thank you for your valuable contribution to the research study.**
