# Supplementary material for: Food fraud and the perceived integrity of European food imports into China
Source: PLoS One. 2018 May 23;13(5):e0195817. doi: 10.1371/journal.pone.0195817 (PMC5965827; doi:10.1371/journal.pone.0195817)
Supplement: S1 Table — Item list and loadings per construct. (DOCX) [file pone.0195817.s002.docx]

**S2 Table: Confirmatory factor analysis. Item list and loadings per construct**

| **Construct and Items** | **Beijing**  **(n = 284)** | **Guangzhou (n = 283)** | **Chengdu (n = 283)** |
| --- | --- | --- | --- |
|  | **λ CFA** | **λ CFA** | **λ CFA** |
| **Food Hazard Concern (FHC)** |  |  |  |
| *To what extend do you agree/disagree that you are worried about each of the following* |  |  |  |
| Adulterated food & drink | 0.933 | 0.887 | 0.855 |
| Inferior quality food & drink | 0.926 | 0.875 | 0.833 |
| Counterfeit food & drink | 0.932 | 0.859 | 0.849 |
| Deteriorated food & drink | 0.929 | 0.867 | 0.823 |
| F & D containing residues of pesticides or vet. drugs | 0.906 | 0.869 | 0.710 |
| Food & drink containing additives | 0.817 | 0.718 | 0.609 |
| Nutritionally imbalanced foods | 0.713 | 0.743 | 0.544 |
| **Benefit of demonstrating Authenticity (BA)** |  |  |  |
| *To what extend do you agree/disagree that demonstrating the authenticity of food/drink will* |  |  |  |
| Improve consumer trust | 0.702 | 0.621 | 0.747 |
| Improve reputation of China | 0.735 | 0.419 | 0.582 |
| Improve trust in the food chain | 0.744 | 0.709 | 0.670 |
| Improve trust in food regulators | 0.764 | 0.670 | 0.721 |
| Improve trust in food and drink products | 0.803 | 0.557 | 0.766 |
| Improve trust in Chinese food and drink manufacturing | 0.772 | 0.641 | 0.644 |
| **Structural & Societal trust SST** |  |  |  |
| *Which organisations or individuals can be trusted to protect the consumer from adulterated and unsafe food & drink* |  |  |  |
| Food Retailers | 0.787 | 0.284 | 0.779 |
| Food Manufactures | 0.769 | 0.792 | 0.898 |
| The media | 0.826 | 0.663 | 0.531 |
| The Government | 0.685 | 0.297 | 0.651 |
| **Authenticity Cues (AC)** |  |  |  |
| *Which of the following do you consider important when making judgements about the authenticity* |  |  |  |
| The barcode | 0.711 | 0.506 | 0.554 |
| The product has certificate of authenticity | 0.715 | 0.571 | 0.613 |
| The product has a tamper proof seal | 0.707 | 0.504 | 0.604 |
| The retailer | 0.708 | 0.487 | 0.470 |
| The packaging | 0.711 | 0.465 | 0.605 |
| The price | 0.586 | 0.497 | 0.614 |
| The nutritional information | 0.668 | 0.366 | 0.598 |
| The brand | 0.681 | 0.562 | 0.534 |
| A product produced & packaged in Europe | 0.608 | 0.523 | 0.537 |
| Country of origin | 0.732 | 0.526 | 0.582 |
| **Attitude (ATT)** |  |  |  |
| *For me ensuring the authenticity of the food/drink I buy is:* |  |  |  |
| Essential | 0.540 | 0.387 | 0.694 |
| Beneficial | 0.760 | 0.635 | 0.754 |
| **Intention (INT)** |  |  |  |
| I plan to purchase food and drinks which have been traced for authenticity | 0.815 | 0.722 | 0.905 |
| I will try to purchase food and drinks which have been traced for authenticity | 0.826 | 0.753 | 0.808 |
| I intend to purchase food and drinks which have been traced for authenticity | 0.797 | 0.781 | 0.750 |
